# Diversity of the *var* gene family of Indonesian *Plasmodium falciparum* isolates

**DOI:** 10.1186/1475-2875-12-80

**Published:** 2013-02-27

**Authors:** Erma Sulistyaningsih, Loeki E Fitri, Thomas Löscher, Nicole Berens-Riha

**Affiliations:** 1 Faculty of Medicine, University of Jember, Jember, 68121, Indonesia; 2 Faculty of Medicine, Brawijaya University, Malang, 65145, Indonesia; 3 Department of Tropical Medicine and Infectious Diseases, University of Munich, Munich, 80802, Germany

**Keywords:** *Plasmodium falciparum*, *var* gene, PfEMP1, ICAM-1

## Abstract

**Background:**

The large polymorphic protein PfEMP1 is encoded by the *var* gene family. PfEMP1 has been shown to play an important role as cytoadherence ligand on the surface of infected erythrocytes and thereby contributes to the distinct pathogenesis of malaria. The study explored the diversity of the DBL1α and DBL2β-C2 domains of the protein from Indonesian *Plasmodium falciparum* field isolates.

**Methods:**

Samples of patients with severe and uncomplicated malaria from two different malaria-endemic areas in Indonesia were collected and DNA directly extracted. Dried blood on filter paper was prepared for RNA extraction. PCR amplicons were either cloned and subsequently sequenced or directly sequenced for analysis on nucleotide and amino acid level. Recently published as well as self-designed primers were used for amplification.

**Results:**

Blood from eight patients was finally used for analysis. Seventy-one different sequences out of over 500 DBL1α sequenced clones were observed, resulting in an average of 8.9 different DBL1α sequences per isolate. The average DBL1α sequence similarity within isolates was similar to between isolates. Phylogenetic analysis demonstrated no clustering of sequences regarding strain or geographical origin. The DBL1α sequences were analysed by distribution of semi-conserved features (cysteine/PoLV1-4 grouping) and classified into six sequence groups. The DBL1α cys2 type was observed in all expressed sequences *in vivo*. Expression of certain DBL sequences implied potential involvement in the pathogenesis. As expected, the DBL2β-C2 domains showed high to moderate homology among each other.

**Conclusion:**

The DBL1α domains of PfEMP1 from clinical Indonesian isolates showed high divergence among same isolates and some similarities with other Asia-Pacific strains. Further investigations of important *var* gene domains with a larger sample size are required to confirm with statistical significance observed associations with severe malaria in Indonesian samples.

## Background

During the erythrocytic cycle, *Plasmodium falciparum* expresses a protein which is exported from the parasite to the surface of the infected erythrocyte (IE) approximately 18 hours post invasion, called *P. falciparum* erythrocyte membrane protein 1 (PfEMP1). This protein has been linked to two key phenomena responsible for the pathology associated with *P. falciparum* infection: cytoadherence of IE and antigenic variation with consequent immune evasion in the host [1-3]. PfEMP1 is a large and polymorphic protein that varies in domain composition and binding specificity. It is encoded by the highly diverse *var* gene family consisting of approximately 60 variable genes per haploid genome of the parasite. Based on chromosomal location, sequence and promoter sequence, the *var* genes are separated into three major groups (A, B, and C) [4].

Despite their diversity, the majority of *var* genes contain a number of conserved motifs. Each *var* gene potentially encodes between two to seven Duffy-binding like (DBL), and cysteine-rich interdomain regions (CIDR). Based on the consensus motifs, DBL domains have been classified into six types; α, β, γ, δ, ε, and x [5]. Specific combinations of domain subtypes are described as short tandem domain cassettes (DC). Rask *et al.* classified the PfEMP1 protein in 628 homology blocks [6]. Due to high conservation in flanking regions and extreme diversity in the inner parts, the amino-terminal DBL domain, DBL1α, has been the main target for diversity studies [7-12].

The extent of *var* gene diversity in different geographic regions has previously been reported [10]. Another study investigating DBL1α sequences of laboratory and field strains showed only 15-20% amino acid conservation [8]. In general, the average level of DBL1α sequence variability within isolates was as great as between isolates [7-9]. However, high overlapping of the *var* gene repertoire in Western Amazon isolates was recently reported [11,12].

The DBL2β-C2 domain mediates binding to the intercellular adhesion molecule 1 (ICAM-1) in several *P. falciparum* isolates. Binding to ICAM-1 seems to play a role in severe disease development. The adhesion to ICAM-1 tended to be higher in patients with cerebral malaria [13]. Further, co-localization of ICAM-1 with parasite sequestration in brain vessels in autopsy samples from cerebral malaria patients [14] and up-regulation of ICAM-1 expression on endothelium during malaria infection was observed [15].

The ICAM-1 binding seems to require the DBL2β-C2 domain including 16 conserved cysteine residues [16-19], as they contain contact residues for ICAM-1. A truncated analysis demonstrated that the critical ICAM-1 binding regions lie between a conserved tryptophan (W) and the first half of the C2 part including the ‘Y motif’. However, the optimal binding activity seems to depend on residues in the first part of the DBLβ [18].

Alignment of ICAM-1 binding DBL2β-C2 domain from three isolates (A4, A4tres and JDP8) revealed significant homology, sharing 16 conserved cysteine residues and a number of conserved hydrophobic amino acid residues. However, multiple alignments of ICAM-1 binding and non-binding DBL2β-C2 domains suggested that the binding region for ICAM-1 is unlikely to lie in a linear sequence stretch within DBL2β-C2 [19].

Recent evidence showed that a conserved ICAM-1-binding epitope lies in a subset of group A PfEMP1 domains called DC4 including DBLβ [20].

Furthermore, an association between *var* gene expression and severity of malaria has been reported. Studies from Brazil and Mali indicated that expression of DBL1α lacking one to two cysteine residues was associated with severe non-cerebral malaria [21,22]. A specific motif of amino acids in DBL1α that is located in a distinct region of receptor interaction was correlated with rosetting and severe malaria (SM) in Ugandan children [23]. Another study found that expression of a specific genotype, referred as *var* D gene with characteristics of the DBLδ domain, was associated with the manifestation of SM [24].

In this study, sequence and motif variability of *var* genes with emphasis on the DBL1α and DBL2β-C2 domains from field isolates collected in two different malaria endemic areas in Indonesia are reported. An attempt was made to elucidate the association of specific DBL domain expression and the manifestation of SM.

## Methods

### Subjects

Malaria patients were enrolled from the Saiful Anwar Hospital, Malang, and from Pelaihari District, Banjarmasin. All patients were informed of the study and written informed consent was obtained. Inclusion criteria were mono-infection with *P. falciparum* by microscopic examination of thin and thick blood smears stained with Giemsa and malaria exposure in Indonesia. Severe and uncomplicated malaria (UM) were categorized based on the clinical and laboratorial criteria, according to WHO diagnosis of malaria patients [25]. Six ml heparinized blood from each patient was collected. A minimum of 10 drops was spotted on Whatman filter paper (Standard-Whatman Cellulose Chromatography paper 3MM). Samples were immediately processed as described below.

The ethical approval was obtained from the Ethical Committee of Medical Research of Brawijaya University, Indonesia.

### RNA isolation and cDNA synthesis

First, cultures of parasitized erythrocytes from the patients were started [26] to gain RNA but abandoned due to laboratory difficulties. Parasites were harvested 18–24 hours post invasion. To investigate the expression in patients directly, total RNA was extracted from dried blood, spotted directly after blood collection on filter paper, using RNeasy Mini Kit (Qiagen). RNA was then reverse transcribed using SuperScript II reverse transcriptase and Oligo-dT primer. The filter paper was dried at room temperature and later stored by −20°C. Samples were shipped on dry ice to the Department of Tropical Medicine and Infectious Diseases in Munich where the extraction was performed.

### Isolation of genomic DNA

Genomic DNA (gDNA) was directly isolated from 600 μl blood samples obtained from malaria patients by DNA Blood kit (Qiagen) in Indonesia and shipped to the Department of Tropical Medicine and Infectious Diseases on dry ice.

### Species differentiation and genotyping

Species differentiation including *P. knowlesi* was performed by PCR as described earlier [27]. All parasite isolates were genotyped by fragment size analysis generated by amplification of the polymorphic single copy genes merozoite surface protein-1 (MSP-1), merozoite surface protein-2 (MSP-2) and glutamate-rich protein (GLURP). PCR was performed as recently described [28]. Diversity among isolates was determined by size difference of amplification products. Genotyping was performed with directly extracted DNA without previous *in vitro* culturing to avoid selection of specific genotypes [29].

### PCR and sequencing of *var* genes

DBL1α domains were amplified from cDNA and gDNA using universal primers αAF and αBR [7]. PCR products were purified using Ultrafree-DA extraction kits (Millipore), cloned into pGEMT-Easy vector (Promega) and transformed into *Escherichia coli* DH5α competent cells (Invitrogen) following manufacturers’ instructions. Recombinants were selected by blue-white colour screening and sequenced using T7 and SP6 primers with Big Dye Terminator v3.1 cycle sequencing kit (Applied Biosystems 3730 sequencer). 20–30 colonies were picked for plasmid isolation from each plate. Three plates per isolate were prepared.

### Classification of the DBL1α sequence tag

The DBL1α sequence tag regions start at a DIGDI motif within homology block (HB) d and end at a PQFLR motif within HB h, according to the classification by Smith *et al.*[5]. The sequences were classified in six sequence groups based on the number of cysteine residues within the tag region and a set of sequence motifs at four positions of limited variability (PoLV 1–4). The positions within the sequences are fixed in relation to four anchor points as proposed by Bull and colleagues. PoLV1 and PoLV4 were defined in relation to the 5′ and the 3′ ends of the sequence, respectively. PoLV2 and PoLV3 are correlated to a “WW” motif. This classification is referred to as cysteine/PoLV sequence grouping [30].

The DBL1α sequence was also characterized by recently described homology blocks (HBs) using the VarDom server [31]. A HB is defined as a member of sequences with similarity to the sequence profile above a certain threshold (9.97) by Rask *et al.*[6]. Smith’s HBs a-j were redefined, i e, as HBd = HB3 and HBh = HB2.

### Identification of DBL2β-C2 domain of each field isolates

Specific primer for the DBL2β-C2 sequence were designed according to several referred sequences of the DBL2β-C2 domain: JDP8 (AY028643), isolate IT4/24/25 (IT-ICAM var) (AY578326), clone A4 strain IT4/25/5 (L42244.1), clone A4tres isolate IT (AF193424) and FCR3var CSA (AJ133811). The amplified fragments from PCR were purified using Ultrafree-DA extraction kits (Millipore), cloned into the pGEMT-Easy vector (Promega), and transformed into *E. coli* JM109 competent cells (Promega) following manufacturers’ instructions. Recombinants were selected by blue-white colour screening and sequenced using T7 and SP6 primers with Big Dye Terminator v3.1 cycle sequencing kit (Applied Biosystems 3730 sequencer). Primer walking was performed to identify the inner sequences of the DBL2β-C2 domain from each isolate (Table 1).

**Table 1:** Primer pairs to amplify DBL2β-C2 domain

**Primer**	**Forward**	**Reverse**
Primers to amplify the whole DBL2β-C2 domain		
DBLβF-R	5′-AGT GTG TTG AAG GAC GTA TGT-3′	5′-CCA AAC ATA TAT CTC TAT AAT CTC C-3′
Primers to amplify the inner sequences of DBL2β-C2 domain (primer walking)		
Pap1_DBLβ	5′-ATG ACT GAA TGG GC(C/A) GAA TG-3′	5′-CAA GAA GTC ATA CAC GGA T-3′
Pap2_DBLβ	5′-ATG ACT GAA TGG GC(C/A) GAA TG-3′	5′-TAC ATT CTG GAT CCT CTT C-3′
Pap3.1_DBLβ	5′-ATG ACT GAA TGG GC(C/A) GAA TG-3′	5′-CGTTGACTTGTGTACCACCA-3′
Pap3.2_DBLβ	5′-ATG ACT GAA TGG GC(C/A) GAA TG-3′	5′-ATG CGT CCT TAT ACT CTG G-3′
Kal1_DBLβ	5′-ATG ACT GAA TGG GC(C/A) GAA TG-3′	5′-TAG TAC CAC CGA TTG AGC GT-3′
Kal2_DBLβ	5′-ATG ACT GAA TGG GC(C/A) GAA TG-3′	5′-TGT TCA TCG TCT TCA CCT T-3′
Kal3_DBLβ	5′-ATG ACT GAA TGG GC(C/A) GAA TG-3′	5′-TAG TAC CAC CGA TTG AGC GT-3′
Kal4_DBLβ	5′-ATG ACT GAA TGG GC(C/A) GAA TG-3′	5′-TAG TAC CAC CGA TTG AGC GT-3′
Kal5_DBLβ	5′-ATG ACT GAA TGG GC(C/A) GAA TG-3′	5′-TAG TAC CAC CGA TTG AGC GT-3′

### Identification of *var* D-like gene

To identify a formerly described *var* D gene, first PCR was conducted using UNIEBP primers [24]. Resulting sequences matched partly with the recently published *var* D sequence. Specific primers (*var*-DF: 5′- AAT TCC T(C/G)A TGA (A/T)TT TAA (G/A)CG -3′ and *var*-DR: 5′- CAC ATA ACA T(T/C)C C(A/T)T TCC A -3′;) were designed according to the ~525 bp resulting sequences (43.7% for selected matrix sequence) [24]. The cycle condition consisted of an initial denaturation at 94°C for 4 min, followed by denaturation at 94°C for 60 sec, annealing at 40° for 60 sec, and elongation at 72°C for 40 sec with 30 cycles. The PCR products were purified using Ultrafree-DA extraction kit and processed for DNA sequencing directly. Each sample was sequenced on both strands.

### Sequence analysis

The nucleotide sequences derived from the field isolates were analysed for sequence similarities by NCBI BLAST [32]. The nucleotide sequences were translated into amino acid sequences using Expasy Translation Tool [33]. Alignment of sequences was performed by ClustalW. Percentage sequence similarity and phylogenetic tree building were carried out using the algorithm in DNASIS MAX version 3.0 based upon a Neighbour-Joining method in a ClustalW program.

### Statistical analysis

The differences between sequence groups were analysed using Fisher’s Exact and *χ*^2^- tests with Yates’ continuity correction for small values. Where applicable, means with standard deviation (SD) were calculated and student’s *t*-test or wilcoxon rank sum (Mann–Whitney) test was applied. Results were considered significant if p-values were <0.05. Software used was stata, version 11.

## Results

Twenty-two Indonesian malaria patients with written informed consent were enrolled in this study, 14 were excluded afterwards due to molecularly confirmed mixed infections [27]. Five severe falciparum malaria patients, hospitalized in the Saiful Anwar Hospital, Malang, and three uncomplicated falciparum malaria patients from Pelaihari District, Banjarmasin, were recruited. All severely ill patients had cerebral malaria, one female was pregnant. Patients had symptoms up to 14 days before they were recruited (Additional file 1). Four SM patients were treated unsuccessfully with chloroquine before admission.

### Clonality of field isolates

For genotyping, the polymorphic regions of MSP-1, MSP-2 and GLURP of eight *P. falciparum* isolates were amplified. Multiple fragments were detected from all allelic families as presented in Additional file 2. Ten different bands in the MAD20 allelic family were found, zero to four in each sample; the fragment size ranged from near 160 bp to approximately 350 bp. The K1 allelic family showed four bands between 180 bp to 350 bp, each sample presented maximum of two. The RO33 allelic family showed two fragments of approximately 160 bp and 180 bp solely in two samples from Kalimantan. By using MSP-2 genotyping, six different bands of the 3D7/IC family were detected, zero to four in each sample ranging from approximately 400 bp to 800 bp. The FC27 family presented 10 different bands, ranging from 270 bp to 600 bp, zero to six per sample. One to two bands in each sample were observed by using GLURP specific primers with seven different bands between 600 bp to 1.1 kb. Amplified bands of MSP-1, MSP-2 and GLURP presenting the same fragment size were considered clonally identical. The multiplicity of infection (MOI) is defined as the minimum number of genetically different parasite lines (clones) present in each sample. The MOI of samples ranged from two to 10 parasite lines for each sample (mean 5, SD 2.8) showing a significantly higher MOI in SM (four to 10 parasite lines) than in UM (two to three, p = 0.02). Samples from Papua showed a higher MOI (six to 10) than from South Kalimantan (two to seven, p = 0.06).

### DBL1α sequence diversity

Genomic DNA derived from eight field isolates, 71 different sequences out of a total of over 500 DBL1α sequences were identified with <95% sequence similarity. On average, 8.9 different DBL1α sequences per isolate were discovered. Accession numbers are given in Additional file 3.

The average DBL1α amino acid sequence similarity within isolates ranged from 46.9 ± 5.5 to 49.4 ± 5.5 and among isolates from 47,0 ± 3.8 to 52.4 ± 3.1. Mean sequence similarity for all samples was 48.3 ± 7.2, separation in origin showed similarity of 48.4 ± 6.6 and 48.5 ± 6.4 for Papua and Kalimantan, respectively. Four DBL1α sequences were shared between two different isolates, the corresponding isolates came each from the same geographical area, Three sequences were found in isolates from Kalimantan (Kal4.C6 and Kal5.C1; Kal4.B1 and Kal5.A4; Kal1.B7 and Kal5.C3) and one was observed in isolates from Papua (Pap2.A2 and Pap3.A1). In comparison with published sequences, only two sequences from Papua (Pap1.13 and Pap3.C8) showed a >95% similarity with isolates from India and Solomon Islands, respectively (Table 2). Phylogenetic analysis demonstrated that there was no clustering of sequences regarding strain or geographical origin (Additional file 4).

**Table 2:** Amino acid sequence similarities of DBL1α in Indonesian isolates compared to sequences from other countries

	**Pap1**	**Pap2**	**Pap3**	**Kal1**	**Kal2**	**Kal3**	**Kal4**	**Kal5**	**Kenya**	**Gabon**	**India**	**PNG-1**	**PNG-2**	**Philippines**	**Solomon**	**Amazonas**	
Pap1	**48.5 ± 6.4**	48.4 ± 6.6	48.6 ± 6.3	48.5 ± 6.1	48.4 ± 6.2	48.3 ± 6.3	48.9 ± 6.6	48.1 ± 6.7	47.3 ± 6.1	54.6 ± 6.7	50.8 ± 14.0	52.7 ± 4.3	55.4 ± 8.6	48.2 ± 11.8	46.7 ± 9.4	44.2 ± 4.0	Pap1
Pap2	0	**48.5 ± 6.4**	48.6 ± 6.7	48.4 ± 6.2	48.2 ± 6.6	47.9 ± 6.9	49.0 ± 7.0	47.8 ± 7.3	48.4 ± 4.1	54.8 ± 4.5	48.4 ± 2.5	50.1 ± 2.0	53.6 ± 3.8	45.1 ± 6.0	45.4 ± 6.1	45.2 ± 5.1	Pap2
Pap3	0	1	**48.9 ± 6.3**	48.6 ± 5.9	48.6 ± 6.1	48.6 ± 6.2	49.2 ± 6.6	48.2 ± 6.8	47.0 ± 3.8	53.9 ± 6.9	47.8 ± 11.6	51.6 ± 2.7	52.9 ± 6.4	52.8 ± 16.6	55.9 ± 22.4	44.9 ± 7.5	Pap3
Kal1	0	0	0	**48.5 ± 5.7**	48.4 ± 5.7	48.3 ± 5.6	49.0 ± 6.3	48.1 ± 6.3	49.4 ± 8.1	51.0 ± 5.1	44.5 ± 3.4	50.1 ± 2.2	53.7 ± 4.8	42.1 ± 3.8	41.9 ± 3.9	44.7 ± 6.4	Kal1
Kal2	0	0	0	0	**48.3 ± 5.9**	48.1 ± 5.8	49.1 ± 6.5	47.8 ± 6.6	47.3 ± 4.5	54.0 ± 3.6	47.5 ± 5.8	49.5 ± 3.3	55.6 ± 5.8	44.3 ± 8.4	44.1 ± 7.0	43.1 ± 5.9	Kal2
Kal3	0	0	0	0	0	**46.9 ± 5.5**	49.2 ± 6.7	47.3 ± 7.0	52.4 ± 3.1	54.6 ± 5.5	51.1 ± 2.6	53.5 ± 4.2	48.4 ± 2.6	49.0 ± 1.6	48.9 ± 0.9	53.6 ± 8.4	Kal3
Kal4	0	0	0	0	0	0	**49.4 ± 6.8**	48.7 ± 7.1	51.2 ± 3.0	52.0 ± 3.3	47.8 ± 3.1	51.0 ± 4.4	54.5 ± 4.2	47.7 ± 6.9	47.9 ± 7.7	46.3 ± 2.8	Kal4
Kal5	0	0	0	1	0	0	2	**47.4 ± 7.4**	47.6 ± 3.4	55.1 ± 5.5	48.0 ± 4.5	52.1 ± 2.6	549 ± 7.0	44.3 ± 3.1	44.7 ± 3.2	48.7 ± 12.9	Kal5
Kenya	0	0	0	0	0	0	0	0	***	41.1	44.2	51.9	44.2	46.5	46.5	43.4	Kenya
Gabon	0	0	0	0	0	0	0	0		***	60.6	52,0	63.8	44.9	44.9	38.6	Gabon
India	1	0	0	0	0	0	0	0			***	48.3	61.7	44.2	45	40.8	India
PNG-1	0	0	0	0	0	0	0	0				***	52.9	44.8	44.5	44.5	PNG-1
PNG-2	0	0	0	0	0	0	0	0					***	40.4	37.5	37.5	PNG-2
Philippines	0	0	0	0	0	0	0	0						***	75,0	51.7	Philippines
Solomon	0	0	1	0	0	0	0	0							***	50.9	Solomon
Amazonas	0	0	0	0	0	0	0	0								***	Amazonas
	Pap1	Pap2	Pap3	Kal1	Kal2	Kal3	Kal4	Kal5	Kenya	Gabon	India	PNG-1	PNG-2	Philippines	Solomon	Amazonas	
	Pap1	Pap2	Pap3	Kal1	Kal2	Kal3	Kal4	Kal5	Kenya	Gabon	India	PNG-1	PNG-2	Philippines	Solomon	Amazonas	
Pap1	**48.5 ± 6.4**	48.4 ± 6.6	48.6 ± 6.3	48.5 ± 6.1	48.4 ± 6.2	48.3 ± 6.3	48.9 ± 6.6	48.1 ± 6.7	47.3 ± 6.1	54.6 ± 6.7	50.8 ± 14.0	52.7 ± 4.3	55.4 ± 8.6	48.2 ± 11.8	46.7 ± 9.4	44.2 ± 4.0	Pap1
Pap2	0	**48.5 ± 6.4**	48.6 ± 6.7	48.4 ± 6.2	48.2 ± 6.6	47.9 ± 6.9	49.0 ± 7.0	47.8 ± 7.3	48.4 ± 4.1	54.8 ± 4.5	48.4 ± 2.5	50.1 ± 2.0	53.6 ± 3.8	45.1 ± 6.0	45.4 ± 6.1	45.2 ± 5.1	Pap2
Pap3	0	1	**48.9 ± 6.3**	48.6 ± 5.9	48.6 ± 6.1	48.6 ± 6.2	49.2 ± 6.6	48.2 ± 6.8	47.0 ± 3.8	53.9 ± 6.9	47.8 ± 11.6	51.6 ± 2.7	52.9 ± 6.4	52.8 ± 16.6	55.9 ± 22.4	44.9 ± 7.5	Pap3
Kal1	0	0	0	**48.5 ± 5.7**	48.4 ± 5.7	48.3 ± 5.6	49.0 ± 6.3	48.1 ± 6.3	49.4 ± 8.1	51.0 ± 5.1	44.5 ± 3.4	50.1 ± 2.2	53.7 ± 4.8	42.1 ± 3.8	41.9 ± 3.9	44.7 ± 6.4	Kal1
Kal2	0	0	0	0	**48.3 ± 5.9**	48.1 ± 5.8	49.1 ± 6.5	47.8 ± 6.6	47.3 ± 4.5	54.0 ± 3.6	47.5 ± 5.8	49.5 ± 3.3	55.6 ± 5.8	44.3 ± 8.4	44.1 ± 7.0	43.1 ± 5.9	Kal2
Kal3	0	0	0	0	0	**46.9 ± 5.5**	49.2 ± 6.7	47.3 ± 7.0	52.4 ± 3.1	54.6 ± 5.5	51.1 ± 2.6	53.5 ± 4.2	48.4 ± 2.6	49.0 ± 1.6	48.9 ± 0.9	53.6 ± 8.4	Kal3
Kal4	0	0	0	0	0	0	**49.4 ± 6.8**	48.7 ± 7.1	51.2 ± 3.0	52.0 ± 3.3	47.8 ± 3.1	51.0 ± 4.4	54.5 ± 4.2	47.7 ± 6.9	47.9 ± 7.7	46.3 ± 2.8	Kal4
Kal5	0	0	0	1	0	0	2	**47.4 ± 7.4**	47.6 ± 3.4	55.1 ± 5.5	48.0 ± 4.5	52.1 ± 2.6	549 ± 7.0	44.3 ± 3.1	44.7 ± 3.2	48.7 ± 12.9	Kal5
Kenya									-	41.1	44.2	51.9	44.2	46.5	46.5	43.4	Kenya
Gabon										-	60.6	52,0	63.8	44.9	44.9	38.6	Gabon
India											-	48.3	61.7	44.2	45	40.8	India
PNG-1												-	52.9	44.8	44.5	44.5	PNG-1
PNG-2													-	40.4	37.5	37.5	PNG-2
Philippines														-	75,0	51.7	Philippines
Solomon															-	50.9	Solomon
Amazonas																-	Amazonas
	Pap1	Pap2	Pap3	Kal1	Kal2	Kal3	Kal4	Kal5	Kenya	Gabon	India	PNG-1	PNG-2	Philippines	Solomon	Amazonas	

The table shows in the right upper half means and standard deviations of similarities of pair wise alignments of sequences. All sequences identified per parasite are compared against each other. Several polypeptide sequences from other areas were selected for comparison. The bold numbers in the diagonal show the intra-parasite sequence similarities in the Indonesian samples. In the upper left side, the number of shared sequences (>95% similarity) between the parasite lines are presented. (Accession numbers for sequences used: India: AF404253; Solomon Islands: AY054815; Kenya-Kilifi: AM115931; Papua New Guinea, Madang, Amele (PNG-1): DQ134266; Papua New Guinea (PNG-2): EU787882; Gabon-Bakoumba: DQ135170; Philippines: AY054892).

### Distribution motifs in DBL1α sequences

**Figure 1:**
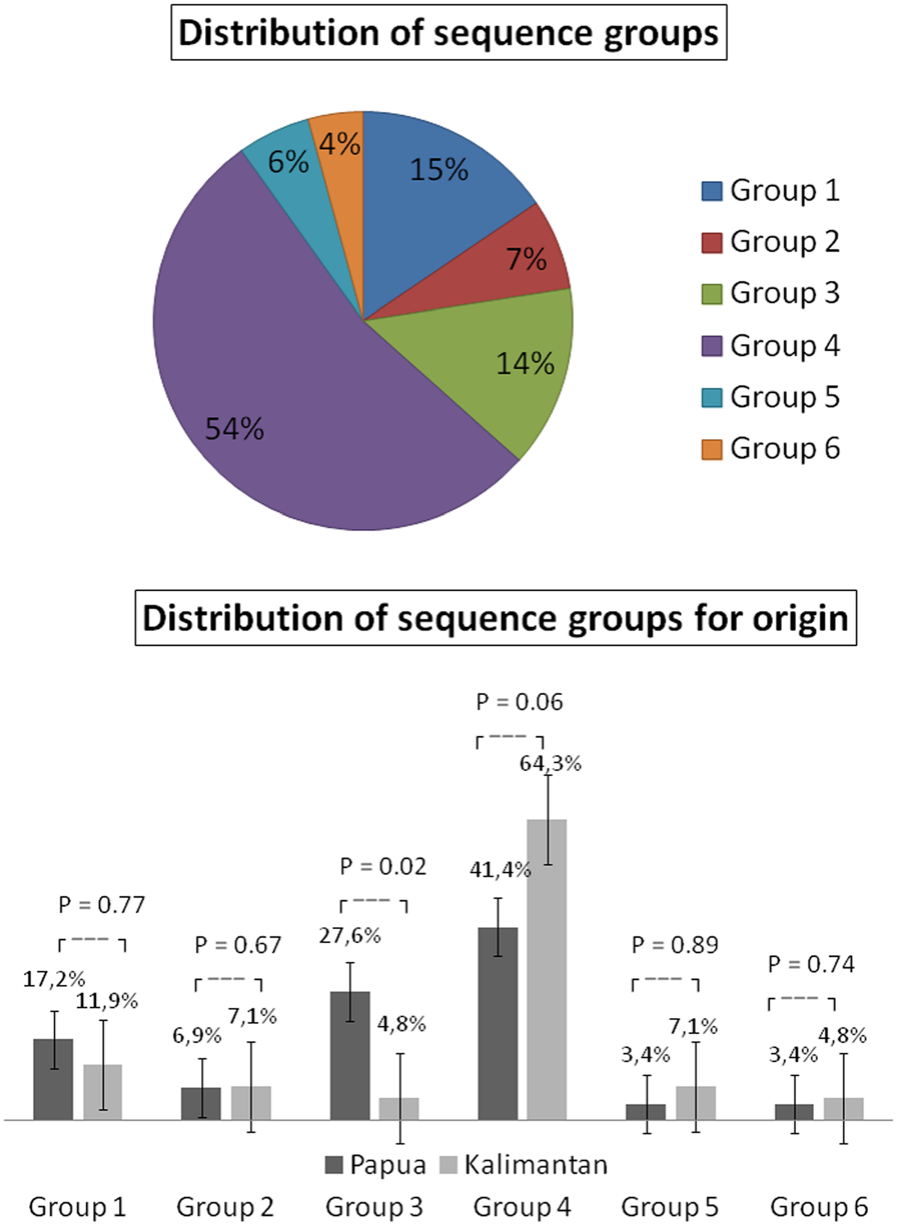
**Distribution of DBL1α sequences from gDNA into six sequence groups based on Cystein/PoLV classification.** Upper part: The pie chart represents the overall distribution of sequence groups in all 71 sequences from eight isolates. Lower part: Black bars represent DBL1α sequences from isolates from Papua. Grey bars represent DBL1α sequences from Kalimantan. There is statistical evidence for a difference in distribution between the two areas. Group 3 is more prevalent in isolates from Papua. Group 4 is the most prevalent in both areas.

Analysis of the six sequence groups of DBL1α showed group 4 as the most prevalent. Stratification in origin indicated strong evidence for a difference in group 3 (p = 0.02) and group 4 (0.06). Two third of the Kalimantan sequences belonged to group 4, whereas most of the Papua sequences belonged to group 3 (Figure 1). All sequences of group 3 were obtained from severe cases, otherwise the distribution of sequence groups between isolates causing UM and SM was equal (p = 0.48) (Additional file 5).

**Figure 2:**
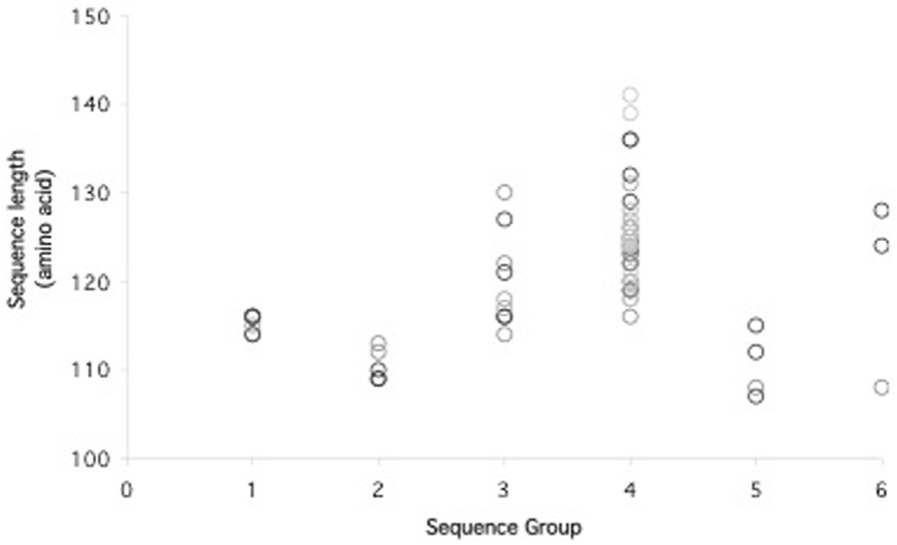
**Association between PoLV motifs and distinct sequence length distribution.** The graph shows the association between PoLV motifs and distinct sequence length. Sequence group 1, 2 and 5 were associated with short sequences. Sequence group 1 contains MFK* at PoLV1 and sequence groups 2 and 5 contain *REY at PoLV2.

**Figure 3:**
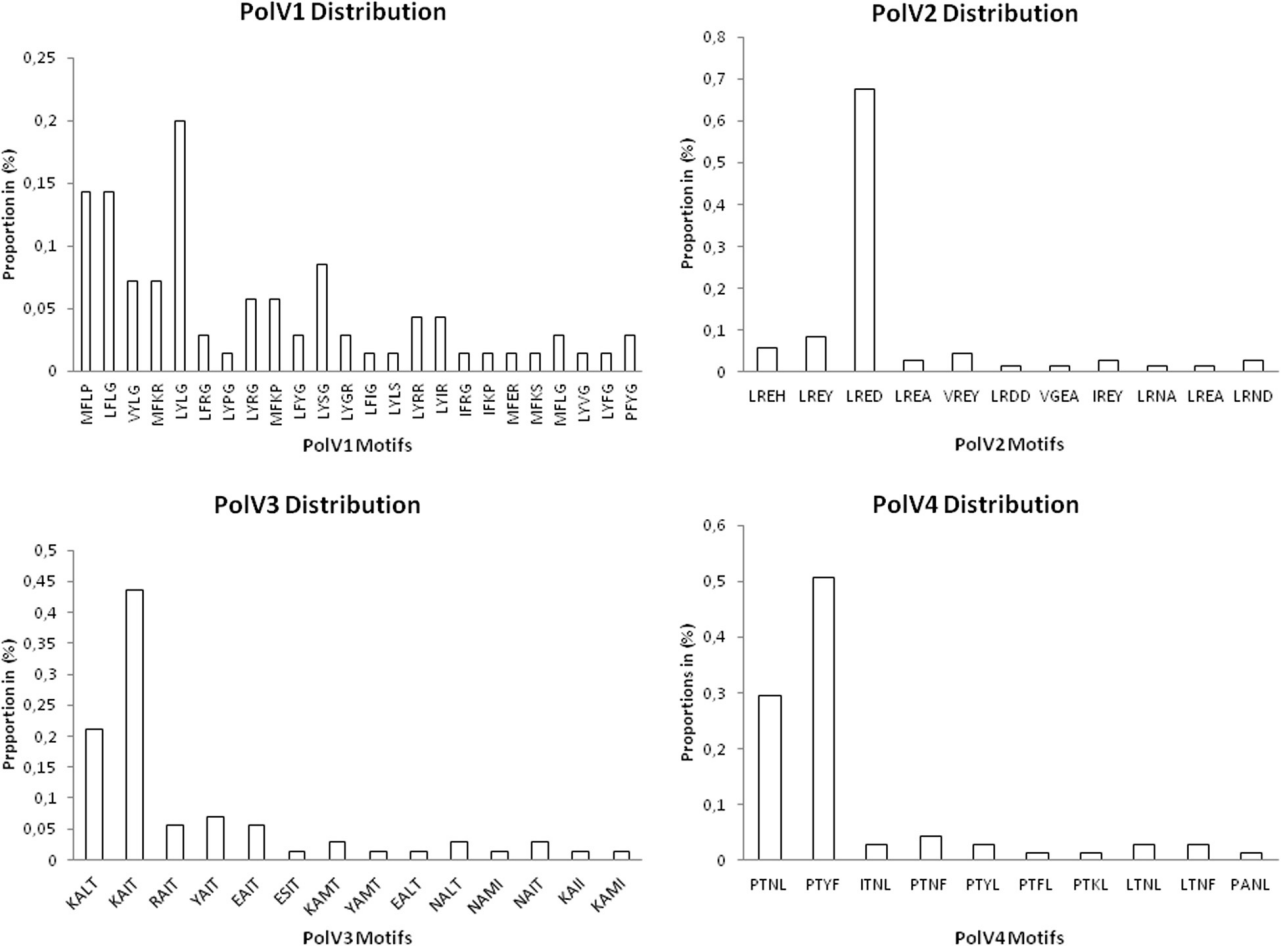
**Distribution of PoLV1-4 motifs in DBL1α from gDNA.** The 71 sequences were classified in six sequence groups based on the number of cysteine residues within the tag region and a set of sequence motifs at four positions of limited variability (PoLV 1–4). This classification is referred to as cysteine/PoLV sequence grouping [30]. In each group, one major motif was found, i.e. LYLG in PoLV1, LRED in PoLV2, KAIT in PoLV3 and PTYF in PoLV4.

Despite the divergence, sequence analysis showed conservation of certain residues in DBL1α domain. The conserved regions were interspersed with variable blocks and varied in both length and sequence. Similar to the report by Bull and colleagues [30] concerning the association between PoLV motifs and distinct sequence length distributions, sequences containing the MFK* at PoLV1 or the *REY at PoLV2 were associated with short sequences (Figure 2). One motif in each PoLV was revealed as the major motif, i e, LYLG in PoLV1, LRED in PoLV2, KAIT in PoLV3 and PTYF in PoLV4 (Figure 3). Some motifs were detected solely in isolates causing SM or UM (Additional file 5).

Analysis of homology blocks defined by Rask *et al.*[6] using varDom server showed that the DBL1α sequences stretched from the most prevalent HB3 to HB2, including subdomain S2b ad S2c. The presence of HB36 in all sequences containing four cysteine residues (cys4) but absence in all sequences containing two cysteine residues (cys2) was also observed as described by Rask and colleagues. HB60 was found in all cys2 sequences and in 11 (21.6%) of 51 cys4 sequences. The same percentage of cys4 sequences presented also HB88 that was absent in cys2 sequences. HBs 2, 3, 5, 14, 54, 60, 64, and 131 were found in both sequence types. HB36, HB79, and HB88 could only be detected in cys4 sequences (Additional files 6 and 7).

### Expressed *var* DBL1α sequence

In only five (four SM, one UM) of eight samples, an expressed DBL1α sequence extracted from filter paper was found using αAF and αBR primers. One of the SM sample was cultivated, synchronized by sorbitol and RNA extracted in trophozoite stage; nine DBL1α sequences were amplified from the cultured cDNA. Three sequences showed similarities of 97-99%, differing minimally in HB5 and 14 and were considered as the same. MOI of the sample was 6.

Cysteine/PoLV classification analysis of expressed sequences from filter paper showed group 1 and 3 with two cysteine residues (cys2) only, consistent with the presence of HB 60 and absence of HB 36 (Additional file 8).

### Identification of DBL2β-C2 domain from field isolates

Ten different sequences out of 37 sequenced clones were identified. From each sample, at least one DBL2β-C2 sequence was found; Pap2 and Pap3 presented two different DBL2β-C2 sequences. Accession numbers are given in Additional file 3.

**Figure 4:**
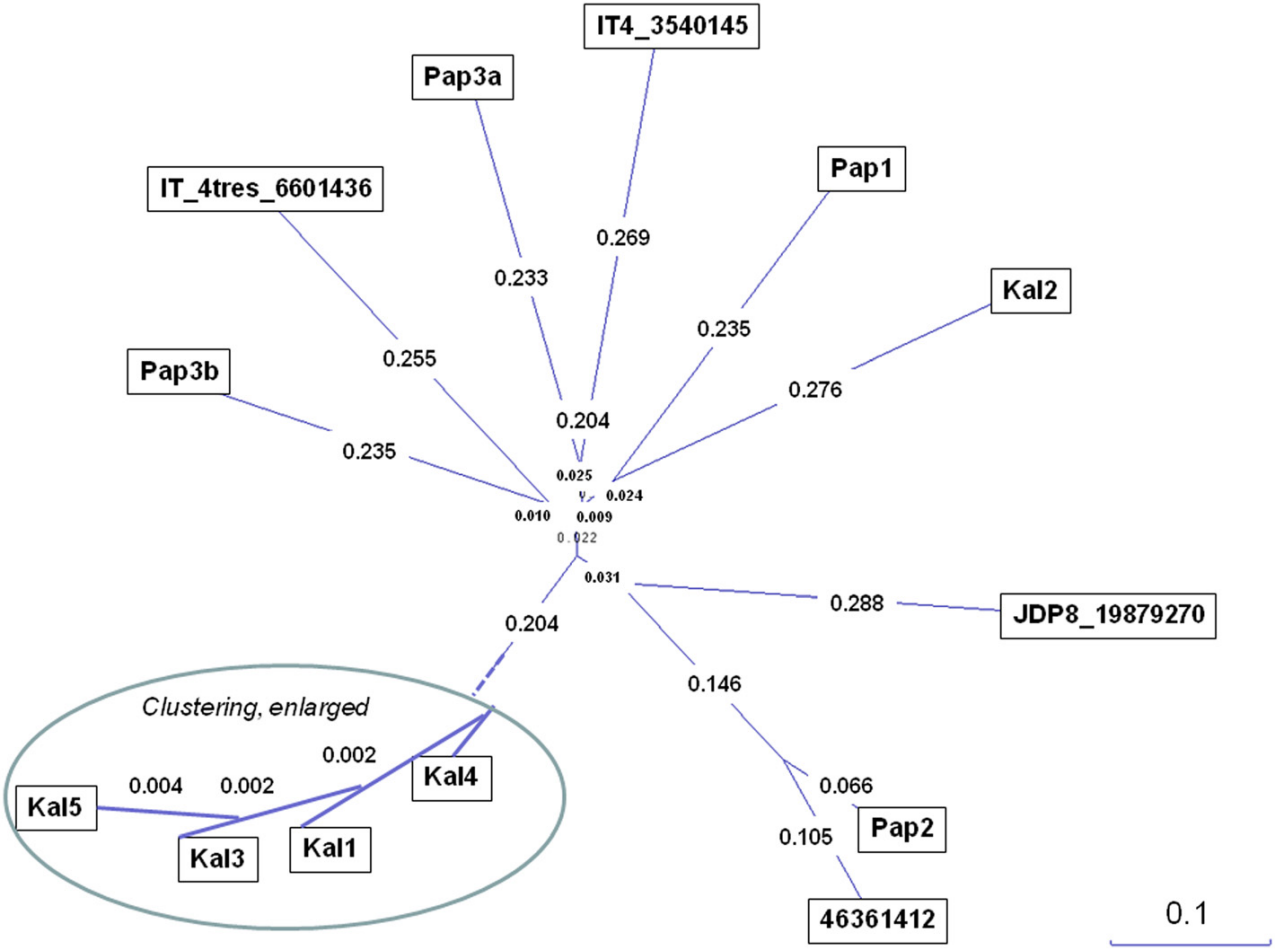
**Unrooted phylogram of DBL2β-C2 sequences from Indonesian field isolates and other global isolates using Neighbour-Joining method.** DBLβ-C2 sequences from three different samples (Kal1, Kal3, and Kal5) presented more than 99% sequence similarity. Kal2 showed low homology with the other Kalimantan isolates. Isolates from Papua were diverse. The amino acid analysis showed 96% identity with the PfEMP1 of IT4/25/5 (ID 46361412; acc no. AY578326.1) strain for Pap2.

DBL2β-C2 sequences from three different samples (Kal1, Kal3, and Kal5) presented more than 99% sequence similarity; all three samples were originated from the same geographical area but had different clinical manifestations (Figure 4). Nucleotide analysis using NCBI database demonstrated 76–84% identity with DBL2β domain of PfEMP1 from distinct *P. falciparum* isolates except for Pap2 with 98% identity with PfEMP1 of IT4/25/5 strain. The amino acid analysis also showed 96% identity with the PfEMP1 of IT4/25/5 (ID 46361412; acc no. AY578326.1) strain for Pap2 but a range of 44–53% identity with the PfEMP1 proteins of several *P. falciparum* field isolates.

As previously described by Smith and colleagues [16], the DBL2β-C2 domains except of Kal2.DBL2β shared many similar features including 16 cysteine residues and blocks of highly conserved amino acids which are flanked by more extensive polymorphic regions (Additional file 9). Compared to other DBL2β-C2 sequences, Kal2.DBL2β-C2 sequence was different; it lacked several conserved cysteine residues, including C6, C8-C11 of DBL2β domain and C13, C15 and C16 of C2 domain. However, the sequence exhibited the ‘Y motif’ within C2 domain, which was reported to play an important role in binding function of DBL2β-C2 domain. Some DBL2β-C2 sequences also revealed little differences such as one more or less cysteine residue, but all sequences had the ‘Y motif’ within the C2 domain.

Further analysis using varDom 1.0 server showed characteristics of the DBL2β domain for all sequences, though Kal2 was classified as un-type DBL domain (Additional file 10). HB analysis of the DBL2β domain demonstrated the presence of five major HBs (HB1-5) in all sequences (Additional file 11). Moreover, HB53 in subdomain S1, HB189 in subdomain S2a, HB59 in subdomain S3a and HB61 in subdomain S3b appeared as HB characteristics of the DBLβ domain.

### Identification of *var* D-like gene

**Figure 5:**
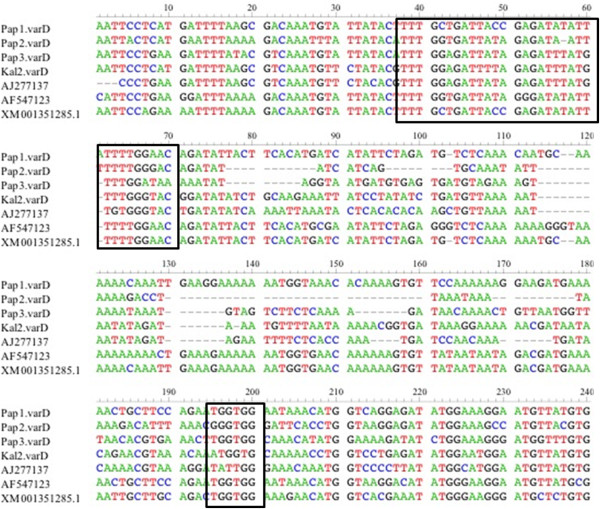
**Multiple alignment of specific *var *D-like sequences.** Specific primers designed for the Indonesian *var*-D like sequence resulted in sequences corresponding to different sequence types including the DBLγ domain of PfEMP-1 isolated from 3D7 [XM_001351285] and patients in association with PAM [AF547123]. Sequence identity was 29.0, 27.8, 27.0 and 19.3% for Kal2, Pap3, Pap1 and Pap2, respectively, with the previously reported *var* D gene (AJ277137). Black boxes: conserved motifs containing of six to 23 nucleotides.

Amplification using UNIEBP primers from genomic DNA resulted in multiple fragments ranging from nearly 250 bp to 1 kb in all samples. A fragment of 525 bp was observed in all samples. Specific primers based on this fragment were designed as described previously [24]. The primers yielded multiple fragments from genomic DNA of all eight samples, but generated a single ~237 bp fragment of cDNA sequences from four SM patients only. The negative SM sample was the same negative with αAF and αBR primers. Sequence identity to the proposed *var* D gene [24] was 29.0, 27.8, 27.0 and 19.3% for *var* D-like sequence of Kal2, Pap3, Pap1 and Pap2, respectively (Figure 5). Sequences highly matched (80-94% identity) with the DBLγ domain of PfEMP1 from several isolates. However, analysis of homology blocks by using varDom 1.0 server presented homology block (HB) characteristics of DBLγ and DBLδ (Additional file 12). The only conserved structures were HB3 and HB5, one sequence shared HB86 with the proposed *var* D gene (AJ277137).

## Discussion

This study analysed *var* genes of *P. falciparum* field isolates derived from two different malaria-endemic areas in Indonesia. Consistent with previous studies on *var* gene diversity [3,5,7,8], the results demonstrated similar variability of DBL1α sequences within and between isolates. This implies that the level of diversity represents the overall DBL1α heterogeneity that exists within each isolate. Regional clustering and conservation like in Amazon isolates was not observed [11,12].

Genotyping results indicated the presence of multiple clones, ranging from two to 10 in each isolate. Patients with SM had significantly more different genotypes than patients with UM. Samples from Papua showed more different clones than those from South Kalimantan. Papua province is an area with stable malaria transmission and a high endemicity class with PfPR_2-10_ ≥ 40% [34] resulting in more than one infection per year per individual [35]. In contrast, South Kalimantan Province is a malaria-endemic area with moderate endemicity (5% < PfPR_2-10_ < 40%) [34]. Some studies reporting the relationship between the mean number of genotypes per infected person and transmission intensity suggested that an increase in transmission level is proportional to parasite clonality per host [36] and *vice versa*[37,38].

Barry and colleagues reported on the global diversity in *P. falciparum var* genes from different geographic regions. The diverse repertoire showed spatial structuring but minimal overlap in the *var* gene repertoire among isolates from different regions, indicating a global distribution of patterns [39]. Data in this study demonstrate little overlap of field isolates from geographically close regions; only four sequences shared similar sequences in isolates from the same area. This is in contrast to the high conservation found in Amazon region mentioned above. As only 8.9 different sequences per isolate were detected, only 15% of the *var* gene repertoire was analysed, multi-clonality neglected. Homologies or further differences might be missed.

86% of all sequenced genes showed >95% similarity to one of the 71 different sequences presented. They belonged each to the same isolate and were considered the same gene. It is possible that some of these sequences were different genes with very similar sequences. However, this would only enhance the homology rate within an isolate not amongst others. One reason for the limited number of different sequences might be sequence variation at the primer sites in Indonesian samples.

Further analysis using cysteine/PoLV grouping on the distribution motif of DBL1α sequence tag in association with the geographical origin revealed a similar sequence distribution between isolates, suggesting that although extremely diverse, DBL1α shares common feature among different geographical origins. Concurred with a previous study which reported a similar distribution of relative number of genomic sequences in each sequence group between field isolates and laboratory isolate 3D7 [40]. The distribution of sequence groups and HBs of genomic DNA among isolates was in general equal, indicating that DBL1α shares common sequences among different clinical categories. Interestingly, group 3 sequences were only found in isolates having caused severe malaria. However, the association lost significance after controlling for origin of the samples; also gDNA lacks information about expression during time of infection. The majority of sequences from both clinical categories belonged to sequence group 4 with four cysteine residues, classified as normal number of cysteine in DBL1α as described previously [30].

All expressed sequences had two cysteine residues and belonged to sequence group 3 or 1, in accordance with a report by Kyriacou and colleagues in Malian children [21]. However, sample size was too low to differentiate between UM and SM cases.

In this study, the DBL2β-C2 domain of Indonesian *P. falciparum* field isolates was identified. Similar to the previous reports [16,19], multiple alignment analysis showed that the DBL2β-C2 sequences shared many similar features, including 16 conserved cysteine residues (C1-C16) and highly conserved amino acid blocks flanked by more extensive polymorphic regions. A clustering in the Kalimantan isolates was observed whereas the isolates from Papua were more diverse (Figure 4). As only one to two different sequences per isolate were analysed - for further experiments, conclusions are limited.

Ariey *et al.* reported a specific sequence called *var* D gene that was expressed in SM. To follow that finding, UNIEBP primers were used. An internal specific primer targeting on *var* D-like gene according to the amplified fragments was designed. The amplicons derived from RNA corresponded partly to the *var* D gene reported previously [24] but closely matched with the DBLγ domain of PfEMP-1 isolated from 3D7 and patients with placenta-associated malaria from various origins (Figure 5). According to Ariey *et al.*, the *var* D gene possessed characteristics of the DBLδ domain, while sequences in this study corresponded to DBLγ domain of PfEMP-1. Further analysis found three conserved sequence motifs containing six to 23 nucleotides. Analysis of homology blocks by using varDom server presented homology block (HB) characteristics of DBLγ and DBLδ. As only HB3 to HB5 was covered, the origin of the sequences remains uncertain, either DBLγ or DBLδ is possible. Rask and colleagues discussed recombination between and reminiscences of DBLγ and DBLδ domains, which might explain the results [6]. Interestingly, expressed sequences were only found in SM patients.

The low identity of the sequences in this study with the proposed *var* D gene excludes relatedness. However, the increased prevalence and programmed expression of those sequences containing certain HBs solely in severe malaria implies its possible involvement in pathogenesis of malaria.

## Conclusion

This report on DBL1α *var* gene variety in Indonesian isolates with different endemicity and pathology shows the immense diversity of the gene family. DBLβ-C2 sequences showed regional clustering in Kalimantan isolates and higher conservation than DBL1α as expected. The prevalence of certain sequences solely in transcripts of SM suggests involvement in the pathogenesis. Based on a larger sample size, further characterization of DBL domains from patients with different clinical manifestations is needed for a thorough analysis of DBL domain expression in falciparum malaria.

## Abbreviations

cDNA: Complementary DNA; CIDR: Cysteine-rich interdomain regions; DBL: Duffy binding-like; EBA: Erythrocytes binding antigen; GLURP: Glutamate-rich protein; ICAM-1: Intercellular adhesion molecule-1; MSP: Merozoite surface protein; TM: Trans-membrane

## Competing interests

The authors declare that they have no competing interests.

## Authors’ contributions

ES conceived the study, performed the laboratory work and the statistical analysis, and wrote the first draft of the manuscript. LEF participated in coordination and collection of samples. TL participated in the design and supervision of study. NBR participated in the design, coordination and analysis of the study and wrote the manuscript. All authors have read the final manuscript and agreed with its contents.

## Supplementary Material

Additional file 1 **Characteristics of malaria patients.** Desription: The table shows gender, age, parasitaemia and clinical outcome of the eight Indonesian patients. Click here for file

Additional file 2 **Genotyping results by MSP-1, MSP-2, and GLURP.** Description: The table shows the multiplicity of infection (MOI) of all samples. Severe malaria cases presented with a higher MOI than those with uncomplicated malaria (p = 0.02). Click here for file

Additional file 3 **Accession numbers of DBL1α and DBLβ-C2 sequences.** Description: The table shows the accession numbers for GenBank, isolate ID, sequence ID and gene of the 71 different DBL1α and 10 differennt DBLβ-C2 sequences mentioned in this study. Additionally, 22 DBLa sequences with >95% similarity to one of the 71 sequences of the same isolate were submitted. Click here for file

Additional file 4 **Unrooted phylogram of DBL1α sequence tags from Indonesian field isolates and other global isolates using Neighbour-Joining method.** Description: Sequences derived from genomic DNA and cDNA of isolates with severe and uncomplicated malaria. Four pairs of DBL1α sequences showed >95% similarity. Sequences from Kal4.C62 and Kal5.C1, Kal4.B12 and Kal5.A4, Kal1.B7 and Kal5.C3 and Pap2.A2 and Pap3.A11 were detected in two different isolates that came from the same geographical area. Two sequences from Papua (Pap1.13 and Pap3.C8) showed a >95% similarity with isolates from India and Solomon Islands. Sequences were clustered in separate groups apart from each other, without regarding strain or geographical origin. Click here for file

Additional file 5 **Distribution of sequence groups and motifs from gDNA within isolates causing severe and uncomplicated malaria.** Description: Upper part: The graph shows the proportional distribution of sequence groups in the two different clinical categories. Sequence group 3 was only found in isolates causing severe malaria. Lower part: The graph shows the distribution of the 71 sequences classified in the cysteine/PoLV sequence grouping [30]. The six sequence groups are based on the number of cysteine residues within the tag region and a set of sequence motifs at four positions of limited variability (PoLV 1-4). Some motifs were detected solely in severe or uncomplicated malaria. Click here for file

Additional file 6 **Distribution of homology blocks in cys4 sequences from DBL1a domain, classification by Rask et al. using VarDom server.** Description: The table shows the distribution of homology blocks (HB) in DBL1a sequences containing four cysteine residues (cys4) using varDom server. Almost all cys4 sequences contained HB3 and HB5 as major homology blocks. HB36 was present in all cys4 sequences whereas HB60 was found only in 15 of 61 (24.6%) of cys4 sequences. Sequences from severe malaria cases, gDNA (white letters). Sequences from severe malaria cases, cDNA culture (orange letters). Sequences from uncomplicated malaria cases, gDNA (black letters). Click here for file

Additional file 7 **Distribution of homology blocks in cys2 sequences from DBL1a domain, classification by Rask et al. using VarDom server.** Description: The table shows the distribution of homology blocks (HB) in DBL1a sequences containing two cysteine residues (cys2) using varDom server. Almost all cys2 sequences contained HB3 and HB5 as major homology blocks. HB60 was present in all cys2 sequences but no HB36 was found in any cys2 sequence. HB14 was present in both cys4 and cys2 sequences. Sequences from severe malaria cases, gDNA (white letters). Sequences from severe malaria cases, cDNA culture (orange letters). Sequences from severe malaria cases, cDNA filter paper (yellow letters on red). Sequence from an uncomplicated case, cDNA filter paper (yellow letters on black). Sequences from uncomplicated malaria cases, gDNA (black letters). Click here for file

Additional file 8 **Motif distribution of expressed var DBL1α Sequences.** Description: The table shows the distribution of expressed var DBL1α sequence motifs (cysteine/PoLV sequence grouping classification by Bull and colleagues). All sequences extracted from blood from filter paper and therefore expressed in the patient during infection showed group 1-3. Click here for file

Additional file 9 **Characteristic of DBLβ-C2 sequences from field isolates.** Description: The graph shows the characteristic of DBLβ-C2 sequences from Indonesian field isolates. DBLβ domain of Indonesian field isolates shared many similar features including 12 cysteine residues and blocks of highly conserved amino acids flanked by more extensive polymorphic regions. The DBLβ domain is always followed by C2 domain containing four invariant cysteine residues. The conserved cysteine residues in DBLβ-C2 are numbered as C1 - C16. The four loops (loop 1-4) and ‘Y motif’ are indicated by boxes. Click here for file

Additional file 10 **Sequence family classification of DBLβ-C2 domain from field isolates using varDom server.** Description: The table shows the score for sequence family classification of the DBLβ-C2 domain from field isolates using varDom server. All sequences were classified as Duffy-binding domain with a high score, more than 255.3 (threshold value 9.97), and all sequences except for DBLβ-C2 domain of Kal2 isolate were classified as DBLβ domain with a score more than 703.1, Kal2_ DBLβ-C2 domain is a non-typical Duffy binding domain. Click here for file

Additional file 11 **Distribution of five major homology blocks in DBLβ domains of field isolates using VarDom server.** Description: The table shows the distribution of five major homology blocks (HB1 – HB5) in DBLβ domains of field isolates using varDom server. All sequences contained five major homology blocks except for Kal2 where HB1 was absent. Click here for file

Additional file 12 **Distribution of homology blocks among the proposed var D gene using VarDom server.** Description: The table shows the distribution of homology blocks (HB) among the proposed var D gene using varDom server. The two motifs HB3 and HB5 seemed to be conserved features. Only one sequence shared HB86 with the previously described var D gene (AJ277137), where HB86 is mainly found in the DBLγ domain. Other sequences presented either HB82, HB98, HB27 or HB624. HB82 and HB98 are found in both DBLγ and DBLδ, while HB27 is mainly found in DBLδ. Click here for file
