# Supplementary material for: Diversity of the var gene family of Indonesian Plasmodium falciparum isolates
Source: Malar J. 2013 Feb 27;12:80. doi: 10.1186/1475-2875-12-80 (PMC3614516; doi:10.1186/1475-2875-12-80)
Supplement: Additional file 1 — Characteristics of malaria patients. Desription: The table shows gender, age, parasitaemia and clinical outcome of the eight Indonesian patients. [file 1475-2875-12-80-S1.doc]

**Additional Table 1. Characteristics of malaria patients**

| **Sample** | **Sex** | **Age (years)** | **Parasitaemia (%)** | **Clinical Outcome** |
| --- | --- | --- | --- | --- |
| Pap1 | M | 35 | 10 | Cerebral malaria, anaemia, respiratory distress, renal failure |
| Kal1 | M | 50 | 2 | Cerebral malaria, clinical jaundice, anaemia |
| Pap2 | F | 19 | 12.8 | Cerebral malaria, clinical jaundice, severe anaemia, PAM |
| Kal2 | M | 47 | 3 | Cerebral malaria, anaemia |
| Pap3 | M | 51 | 24.6 | Cerebral malaria, clinical jaundice, renal failure, hepatic dysfunction |
| Kal3 | M | 19 | 1 | Mild malaria |
| Kal4 | M | 35 | 1 | Mild malaria |
| Kal5 | M | 55 | 2 | Mild malaria |
