# Supplementary material for: Diversity of the var gene family of Indonesian Plasmodium falciparum isolates
Source: Malar J. 2013 Feb 27;12:80. doi: 10.1186/1475-2875-12-80 (PMC3614516; doi:10.1186/1475-2875-12-80)
Supplement: Additional file 2 — Genotyping results by MSP-1, MSP-2, and GLURP. Description: The table shows the multiplicity of infection (MOI) of all samples. Severe malaria cases presented with a higher MOI than those with uncomplicated malaria (p = 0.02). [file 1475-2875-12-80-S2.doc]

**Additional Table 2.** Genotyping results by MSP-1, MSP-2, and GLURP

| **Sample** | **Number of MSP-1 bands** | | | **Number of MSP-2 bands** | | **Number of GLURP bands** | **MOI^*^** |
| --- | --- | --- | --- | --- | --- | --- | --- |
|  | **MAD20** | **K1** | **RO33** | **3D7/IC** | **FC27** |  |  |
| Pap1 | 4 | 2 | 0 | 1 | 4 | 1 | **6** |
| Kal1 | 1 | 0 | 1 | 2 | 2 | 2 | **4** |
| Pap2 | 4 | 2 | 0 | 3 | 0 | 1 | **6** |
| Kal2 | 3 | 2 | 0 | 1 | 6 | 1 | **7** |
| Pap3 | 4 | 1 | 0 | 4 | 6 | 2 | **10** |
| Kal3 | 0 | 1 | 1 | 1 | 1 | 1 | **2** |
| Kal4 | 2 | 0 | 0 | 0 | 2 | 2 | **2** |
| Kal5 | 1 | 1 | 0 | 1 | 2 | 1 | **3** |
