# Supplementary material for: Diversity of the var gene family of Indonesian Plasmodium falciparum isolates
Source: Malar J. 2013 Feb 27;12:80. doi: 10.1186/1475-2875-12-80 (PMC3614516; doi:10.1186/1475-2875-12-80)
Supplement: Additional file 3 — Accession numbers of DBL1α and DBLβ-C2 sequences. Description: The table shows the accession numbers for GenBank, isolate ID, sequence ID and gene of the 71 different DBL1α and 10 differennt DBLβ-C2 sequences mentioned in this study. Additionally, 22 DBLa sequences with >95% similarity to one of the 71 sequences of the same isolate were submitted. [file 1475-2875-12-80-S3.doc]

**Additional Table 3. Accession numbers (GenBank) for DBLα and DBLβ-C2 sequences**

| Accession Number | Sample | Sequence ID | Gene |
| --- | --- | --- | --- |
| BankIt1602094 Seq1  KC608869 | Pap1 | 1B3 | DBL α |
| BankIt1602094 Seq2  KC608870 | Pap1 | 1A2 | DBL α |
| BankIt1602103 Seq1  KC608871 | Pap1 | 1A3 | DBL α |
| BankIt1602103 Seq2  KC608872 | Pap1 | 1A4 | DBL α |
| BankIt1602103 Seq3  KC608873 | Pap1 | 1A6 | DBL α |
| BankIt1602103 Seq4  KC608874 | Pap1 | 1C3 | DBL α |
| BankIt1602120 Seq1  KC608875 | Kal1 | 2C7 | DBL α |
| BankIt1602120 Seq2  KC608876 | Kal1 | 2C1 | DBL α |
| BankIt1602120 Seq3  KC608877 | Kal1 | 2C2 | DBL α |
| BankIt1602120 Seq4  KC608878 | Kal1 | 2C5 | DBL α |
| BankIt1602120 Seq5  KC608879 | Kal1 | 2C6 | DBL α |
| BankIt1602120 Seq6  KC608880 | Kal1 | 2B1 | DBL α |
| BankIt1602120 Seq7  KC608881 | Kal1 | 2B4 | DBL α |
| BankIt1602120 Seq8  KC608882 | Kal1 | 2B9 | DBL α |
| BankIt1602120 Seq9  KC608883 | Kal1 | 2A1 | DBL α |
| BankIt1602120 Seq10 KC608884 | Kal1 | 2A2 | DBL α |
| BankIt1602120 Seq11 KC608885 | Kal1 | 2A3 | DBL α |
| BankIt1602120 Seq12 KC608886 | Kal1 | 2B11 | DBL α |
| BankIt1603208 Seq1  KC608887 | Kal1 | 2B3 | DBL α |
| BankIt1603208 Seq2  KC608888 | Kal1 | 2B6 | DBL α |
| BankIt1603208 Seq3  KC608889 | Kal1 | 2B7 | DBL α |
| BankIt1603208 Seq4  KC608890 | Kal1 | 2C4 | DBL α |
| BankIt1603208 Seq5  KC608891 | Kal1 | 2B8 | DBL α |
| BankIt1603217 Seq1  KC608892 | Pap2 | 4A1 | DBL α |
| BankIt1603217 Seq2  KC608893 | Pap2 | 4A2 | DBL α |
| BankIt1603217 Seq3  KC608894 | Pap2 | 4A3 | DBL α |
| BankIt1603217 Seq4  KC608895 | Pap2 | 4A4 | DBL α |
| BankIt1603217 Seq5  KC608896 | Pap2 | 4B1 | DBL α |
| BankIt1603217 Seq6  KC608897 | Pap2 | 4C1 | DBL α |
| BankIt1603217 Seq7  KC608898 | Pap2 | 4C2 | DBL α |
| BankIt1603217 Seq8  KC608899 | Pap2 | 4C3 | DBL α |
| BankIt1603217 Seq9  KC608900 | Pap2 | 4C4 | DBL α |
| BankIt1603488 Seq1  KC608901 | Kal2 | 5A1 | DBL α |
| BankIt1603488 Seq2  KC608902 | Kal2 | 5A2 | DBL α |
| BankIt1603488 Seq3  KC608903 | Kal2 | 5A3 | DBL α |
| BankIt1603488 Seq4  KC608904 | Kal2 | 5A4 | DBL α |
| BankIt1603488 Seq5  KC608905 | Kal2 | 5B1 | DBL α |
| BankIt1603488 Seq6  KC608906 | Kal2 | 5B2 | DBL α |
| BankIt1603488 Seq7  KC608907 | Kal2 | 5C10 | DBL α |
| BankIt1603488 Seq8  KC608908 | Kal2 | 5C2 | DBL α |
| BankIt1603488 Seq9  KC608909 | Kal2 | 5C3 | DBL α |
| BankIt1603488 Seq10 KC608910 | Kal2 | 5C4 | DBL α |
| BankIt1603488 Seq11 KC608911 | Kal2 | 5C42 | DBL α |
| BankIt1603493 Seq1  KC608912 | Pap3 | 11A1 | DBL α |
| BankIt1603493 Seq2  KC608913 | Pap3 | 11A2 | DBL α |
| BankIt1603493 Seq3  KC608914 | Pap3 | 11A8 | DBL α |
| BankIt1603493 Seq4  KC608915 | Pap3 | 11A10 | DBL α |
| BankIt1603493 Seq5  KC608916 | Pap3 | 11A11 | DBL α |
| BankIt1603493 Seq6  KC608917 | Pap3 | 11B3 | DBL α |
| BankIt1603493 Seq7  KC608918 | Pap3 | 11B6 | DBL α |
| BankIt1603493 Seq8  KC608919 | Pap3 | 11C4 | DBL α |
| BankIt1603493 Seq9  KC608920 | Pap3 | 11C8 | DBL α |
| BankIt1603738 Seq1  KC608921 | Kal3 | L1E7 | DBL α |
| BankIt1603738 Seq2  KC608922 | Kal3 | L1F7 | DBL α |
| BankIt1603738 Seq3  KC608923 | Kal3 | L1F10 | DBL α |
| BankIt1604010 Seq1  KC608924 | Kal4 | L10A2 | DBL α |
| BankIt1604010 Seq2  KC608925 | Kal4 | L10A3 | DBL α |
| BankIt1604010 Seq3  KC608926 | Kal4 | L10A3.2 | DBL α |
| BankIt1604010 Seq4  KC608927 | Kal4 | L10A4.2 | DBL α |
| BankIt1604010 Seq5  KC608928 | Kal4 | L10A5 | DBL α |
| BankIt1604010 Seq6  KC608929 | Kal4 | L10B1 | DBL α |
| BankIt1604010 Seq7  KC608930 | Kal4 | L10B2 | DBL α |
| BankIt1604010 Seq8  KC608931 | Kal4 | L10B5 | DBL α |
| BankIt1604010 Seq9  KC608932 | Kal4 | L10B22 | DBL α |
| BankIt1604010 Seq10 KC608933 | Kal4 | L10C1 | DBL α |
| BankIt1604010 Seq11 KC608934 | Kal4 | L10C42 | DBL α |
| BankIt1604010 Seq12 KC608935 | Kal4 | L10C62 | DBL α |
| BankIt1604033 Seq1  KC608936 | Kal5 | 14A4 | DBL α |
| BankIt1604033 Seq2  KC608937 | Kal5 | 14B2 | DBL α |
| BankIt1604033 Seq3  KC608938 | Kal5 | 14C1 | DBL α |
| BankIt1604033 Seq4  KC608939 | Kal5 | 14C2 | DBL α |
| BankIt1604033 Seq5  KC608940 | Kal5 | 14C3 | DBL α |
| BankIt1604033 Seq6  KC608941 | Kal5 | 14C4 | DBL α |
| BankIt1604033 Seq7  KC608942 | Kal5 | 14C5 | DBL α |
| BankIt1604033 Seq8  KC608943 | Kal5 | 14C6 | DBL α |
| BankIt1604033 Seq9  KC608944 | Kal5 | 14C7 | DBL α |
| BankIt1604033 Seq10 KC608945 | Kal5 | 14C8 | DBL α |
| BankIt1604051 Seq1  KC608946 | Pap1 | 1c | DBL α |
| BankIt1604051 Seq2  KC608947 | Pap1 | 1.1c | DBL α |
| BankIt1604051 Seq3  KC608948 | Pap1 | 1.12c | DBL α |
| BankIt1604051 Seq4  KC608949 | Pap1 | 132c | DBL α |
| BankIt1604051 Seq5  KC608950 | Pap1 | 1.4c | DBL α |
| BankIt1604051 Seq6  KC608951 | Pap1 | 1.12c | DBL α |
| BankIt1604051 Seq7  KC608952 | Pap1 | 1.22c | DBL α |
| BankIt1604051 Seq8  KC608953 | Pap1 | 131c | DBL α |
| BankIt1604051 Seq9  KC608954 | Pap1 | 132c | DBL α |
| BankIt1604051 Seq10 KC608955 | Pap1 | 134c | DBL α |
| BankIt1604051 Seq11 KC608956 | Pap1 | 135c | DBL α |
| BankIt1604736 Seq1  KC608957 | Pap1 | FPPap1 | DBL α |
| BankIt1604736 Seq2  KC608958 | Pap2 | FPPap2 | DBL α |
| BankIt1604736 Seq3  KC608959 | Pap3 | FPPap3 | DBL α |
| BankIt1604740 Seq1  KC608960 | Kal2 | FPKal2 | DBL α |
| BankIt1604740 Seq2  KC608961 | Kal5 | FPKal5 | DBL α |
| BankIt1604892 Seq1  KC608962 | Pap1 | Pap1 | DBLβ-C2 |
| BankIt1604892 Seq2  KC608963 | Pap2 | Pap2.1 | DBLβ-C2 |
| BankIt1604892 Seq3  KC608964 | Pap2 | Pap2.2 | DBLβ-C2 |
| BankIt1604892 Seq4  KC608965 | Pap3 | Pap3.1 | DBLβ-C2 |
| BankIt1604892 Seq5  KC608966 | Pap3 | Pap3.2 | DBLβ-C2 |
| BankIt1605081 Seq1  KC608967 | Kal1 | Kal1 | DBLβ-C2 |
| BankIt1605081 Seq2  KC608968 | Kal2 | Kal2 | DBLβ-C2 |
| BankIt1605081 Seq3  KC608969 | Kal3 | Kal3 | DBLβ-C2 |
| BankIt1605081 Seq4  KC608970 | Kal4 | Kal4 | DBLβ-C2 |
| BankIt1605081 Seq5  KC608971 | Kal5 | Kal5 | DBLβ-C2 |
