# Supplementary figures and images for: Diversity of the var gene family of Indonesian Plasmodium falciparum isolates
Source: Malar J. 2013 Feb 27;12:80. doi: 10.1186/1475-2875-12-80 (PMC3614516; doi:10.1186/1475-2875-12-80)

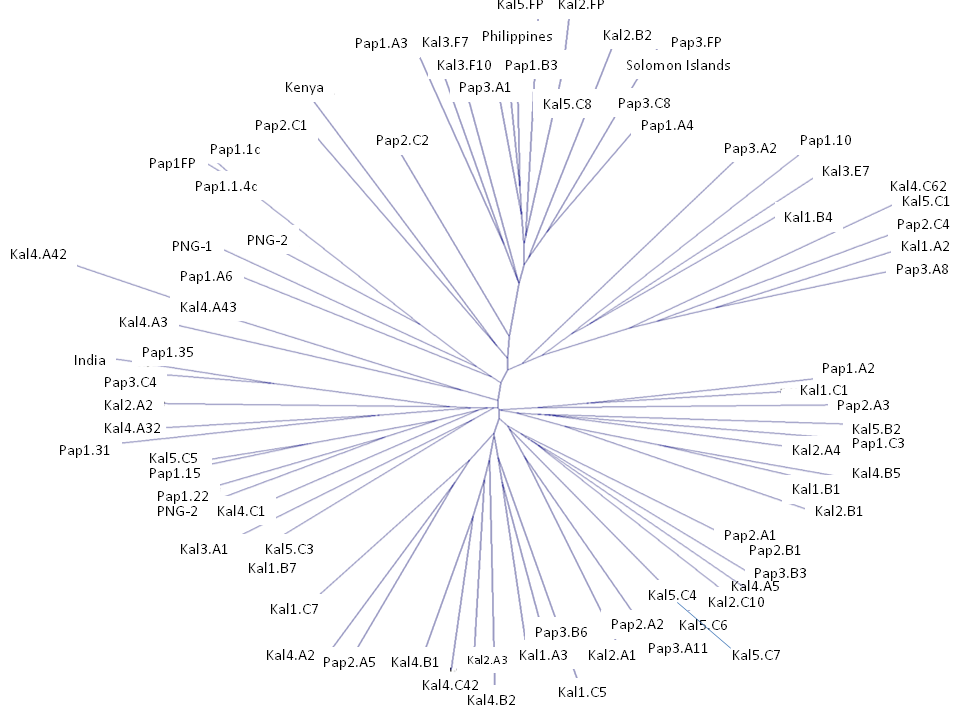

Supplement: Additional file 4 — Unrooted phylogram of DBL1α sequence tags from Indonesian field isolates and other global isolates using Neighbour-Joining method. Description: Sequences derived from genomic DNA and cDNA of isolates with severe and uncomplicated malaria. Four pairs of DBL1α sequences showed >95% similarity. Sequences from Kal4.C62 and Kal5.C1, Kal4.B12 and Kal5.A4, Kal1.B7 and Kal5.C3 and Pap2.A2 and Pap3.A11 were detected in two different isolates that came from the same geographical area. Two sequences from Papua (Pap1.13 and Pap3.C8) showed a >95% similarity with isolates from India and Solomon Islands. Sequences were clustered in separate groups apart from each other, without regarding strain or geographical origin. [file 1475-2875-12-80-S4.tiff]

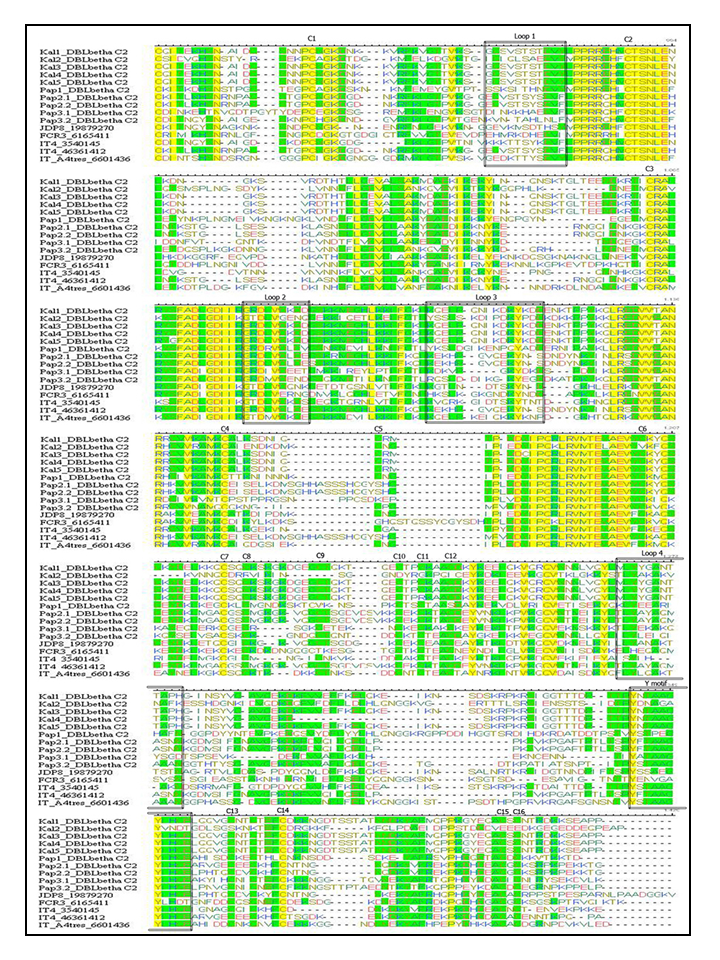

Supplement: Additional file 5 — Distribution of sequence groups and motifs from gDNA within isolates causing severe and uncomplicated malaria. Description: Upper part: The graph shows the proportional distribution of sequence groups in the two different clinical categories. Sequence group 3 was only found in isolates causing severe malaria. Lower part: The graph shows the distribution of the 71 sequences classified in the cysteine/PoLV sequence grouping [30]. The six sequence groups are based on the number of cysteine residues within the tag region and a set of sequence motifs at four positions of limited variability (PoLV 1-4). Some motifs were detected solely in severe or uncomplicated malaria. [file 1475-2875-12-80-S5.tiff]

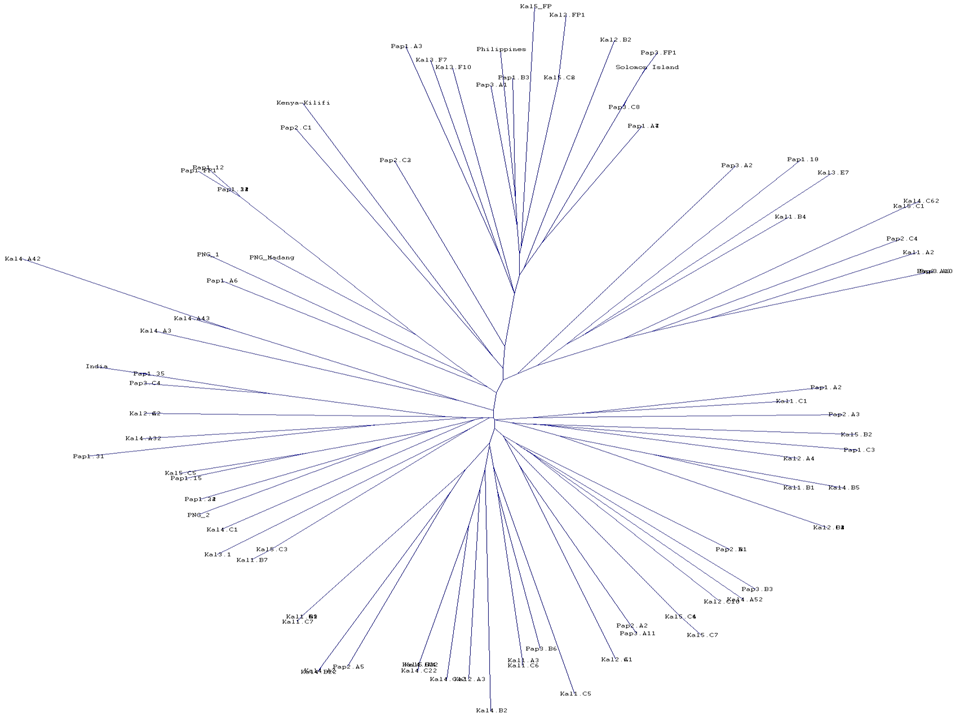

Supplement: Additional file 9 — Characteristic of DBLβ-C2 sequences from field isolates. Description: The graph shows the characteristic of DBLβ-C2 sequences from Indonesian field isolates. DBLβ domain of Indonesian field isolates shared many similar features including 12 cysteine residues and blocks of highly conserved amino acids flanked by more extensive polymorphic regions. The DBLβ domain is always followed by C2 domain containing four invariant cysteine residues. The conserved cysteine residues in DBLβ-C2 are numbered as C1 - C16. The four loops (loop 1-4) and ‘Y motif’ are indicated by boxes. [file 1475-2875-12-80-S9.tiff]
