# Supplementary material for: Diversity of the var gene family of Indonesian Plasmodium falciparum isolates
Source: Malar J. 2013 Feb 27;12:80. doi: 10.1186/1475-2875-12-80 (PMC3614516; doi:10.1186/1475-2875-12-80)
Supplement: Additional file 6 — Distribution of homology blocks in cys4 sequences from DBL1a domain, classification by Rask et al. using VarDom server. Description: The table shows the distribution of homology blocks (HB) in DBL1a sequences containing four cysteine residues (cys4) using varDom server. Almost all cys4 sequences contained HB3 and HB5 as major homology blocks. HB36 was present in all cys4 sequences whereas HB60 was found only in 15 of 61 (24.6%) of cys4 sequences. Sequences from severe malaria cases, gDNA (white letters). Sequences from severe malaria cases, cDNA culture (orange letters). Sequences from uncomplicated malaria cases, gDNA (black letters). [file 1475-2875-12-80-S6.doc]

**Additional Table 4. Distribution of homology blocks (HB) in** **cys4 sequences from DBL1α domain, classification by Rask *et al*. using VarDom server**

| Sequence | Score  HB3 HB5 HB2 | | | Score S2b | | | | Score S2c | | | |
| --- | --- | --- | --- | --- | --- | --- | --- | --- | --- | --- | --- |
|  |  |  |  | HB64 | HB54 | HB14 | HB79 | HB131 | HB60 | HB36 | HB88 |
| Pap1.A2 | 15 | 37.8 |  | 17.8 |  | 34.3 |  |  |  | 38.1 |  |
| Pap1.A6 | 15.2 | 37.1 |  | 13.7 |  | 30.5 |  |  |  | 11.3 |  |
| Pap1.C3 | 15.2 | 35.2 |  | 10.5 |  | 10.0 |  |  |  | 44.5 |  |
| Pap1.15 | 15.2 | 34.1 |  | 19.7 | 11.2 | 31.6 |  |  | 12.1 | 43.7 |  |
| Pap1.22 | 15.2 | 40.4 |  |  | 11.1 | 37.9 |  |  |  | 50.2 |  |
| Pap1.35 | 15.2 | 34.2 |  |  | 15.6 | 25.6 |  | 10.5 | 10.5 | 40.3 |  |
| Pap2.A1 | 15.0 | 39.3 |  |  | 10.8 | 28.8 | 12.4 |  |  | 42.7 | 11.6 |
| Pap2.A2 | 17.9 | 37.6 |  | 12.3 |  | 31.8 | 14.3 | 25.8 |  | 45.4 |  |
| Pap2.A3 | 15.2 | 34.9 |  | 15.0 | 12.8 | 32 |  |  |  | 50.7 |  |
| Pap2.A5 |  | 34.4 |  |  |  | 24.6 |  |  |  | 47.5 |  |
| Pap2.B1 | 17.9 | 39.3 |  |  | 10.8 | 28.8 | 12.4 |  |  | 42.7 | 11.6 |
| Pap3.A11 | 15.0 | 37.6 |  | 12.3 |  | 31.8 | 14.3 | 25.8 |  | 45.4 |  |
| Pap3.B3 | 15.0 | 32.2 |  |  | 13.2 | 18.7 | 13.6 |  |  | 53.6 |  |
| Pap3.B6 | 15.2 | 36.4 |  | 14.9 |  | 29.9 |  | 18.1 |  | 48.2 |  |
| Pap3.C4 | 15.2 | 34.2 | 11.8 |  |  | 26.4 |  |  | 10.5 | 40.3 |  |
| Kal1.A1 | 15.0 | 33.8 | 11.5 |  |  | 20.0 | 15.1 |  | 15.3 | 50.2 |  |
| Kal1.A3 | 15.0 | 37.2 |  | 16.7 |  | 32.0 | 14.5 |  |  | 59.3 | 11.6 |
| Kal1.B1 | 15.0 | 37.0 |  | 13.5 |  | 25.5 |  |  |  | 48.5 | 11.6 |
| Kal1.B4 | 15.0 | 36.8 | 11.5 |  | 10.7 | 17.2 |  |  |  | 27.7 |  |
| Kal1.B7 | 18.1 | 37.0 |  | 14.8 | 18.2 | 33.0 |  |  |  | 45.1 |  |
| Kal1.B6 | 17.9 | 38.7 | 13.4 | 14.6 | 11.5 | 22.8 |  |  | 10.3 | 50.7 |  |
| Kal1.B8 | 18.1 | 40.2 | 13.4 | 15.3 |  | 29.1 | 18.2 |  |  | 33.2 |  |
| Kal1.B9 | 15.0 | 33.8 | 11.5 |  |  | 20.0 | 15.1 |  | 15.3 | 50.2 |  |
| Kal1.C1 | 12.5 | 40.2 |  | 16.9 | 19.1 | 34.6 |  |  |  | 51.4 |  |
| Kal1.C2 | 15.0 | 33.8 |  |  |  | 20.0 | 15.1 |  | 15.3 | 50.2 |  |
| Kal1.C5 | 15.0 | 35.8 |  | 14.7 | 10.3 | 27.0 |  |  |  | 55.5 |  |
| Kal1.C6 | 15.0 | 37.2 |  | 16.7 |  | 32.0 | 14.5 |  |  | 59.3 | 11.6 |
| Kal1.C7 | 15.0 | 33.8 |  |  |  | 20.0 | 15.1 |  | 15.3 | 50.2 |  |
| Kal1.C3 |  | 36.1 |  | 13.7 |  | 24.5 |  |  |  | 38.4 |  |
| Kal1.C4 | 17.9 | 38.0 | 11.5 | 19.7 | 16.6 | 27.0 |  |  |  | 10.2 |  |
| Kal2.A1 | 15.2 | 38.2 | 13.4 | 13.4 | 19.4 | 32.3 |  |  |  | 43.5 |  |
| Kal2.A2 | 15.2 | 35.6 |  |  |  | 30.5 |  |  | 10.4 | 48.2 |  |
| Kal2.A3 | 15.0 | 36.2 |  | 16.8 |  | 27.1 | 13.3 |  |  | 50.8 | 11.6 |
| Kal2.A4 | 15.0 | 35.7 |  |  | 13.1 | 20.2 |  |  |  | 27.1 |  |
| Kal2.B1 | 15.0 | 36.2 |  | 16.6 | 10.0 | 25.7 |  |  |  | 56.2 | 11.6 |
| Kal2.C1 | 15.2 | 38.2 |  | 13.4 | 19.4 | 32.3 |  |  |  | 43.5 |  |
| Kal2.C2 | 15.2 | 35.6 |  |  |  | 30.5 |  |  | 10.4 | 48.2 |  |
| Kal2.C3 | 15.0 | 36.2 |  | 16.6 | 10.0 | 25.7 |  |  |  | 56.2 | 11.6 |
| Kal2.C10 | 15.2 | 30.5 |  | 14.0 | 12.2 | 30.3 |  |  | 16.0 | 51.3 | 11.6 |
| Kal3.A1 | 19.4 | 38.0 | 11.8 |  | 11.5 | 28.6 |  |  |  | 46.0 |  |
| Kal3.E7 | 17.9 | 44.2 |  |  |  | 15.8 |  |  | 10.3 | 15.7 |  |
| Kal4.A2 | 17.9 | 35.6 |  |  |  | 17.0 |  |  |  | 39.4 |  |
| Kal4.A3 | 18.1 | 39.1 |  |  |  | 34.6 |  |  |  | 32.0 |  |
| Kal4.A32 | 18.1 | 46.5 |  | 14.8 | 13.6 | 39.8 |  |  | 12.3 | 35.5 |  |
| Kal4.A42 | 15.2 |  |  | 14.6 | 13.0 | 34.7 | 16.8 |  |  | 30.4 |  |
| Kal4.A43 | 18.1 | 39.2 |  | 14.6 | 13.0 | 34.7 | 16.8 |  |  | 37.2 |  |
| Kal4.A52 |  | 35.2 | 11.8 | 13.7 |  | 32.3 |  | 10.1 |  | 46.4 | 11.6 |
| Kal4.B2 | 17.9 | 38.7 |  | 13.8 |  | 26.2 | 18.2 |  |  | 35.4 | 11.6 |
| Kal4.B5 | 17.9 | 31.9 |  | 13.5 |  | 25.5 |  |  |  | 55.0 |  |
| Kal4.B12 | 15.0 | 35.3 |  | 14.1 |  | 28.3 | 13.4 |  |  | 47.5 | 11.6 |
| Kal4.B22 | 17.9 | 33.2 |  |  |  | 17.0 |  |  |  | 39.4 |  |
| Kal4.C1 | 18.1 | 38.4 |  | 11.8 |  | 34.4 |  | 12.9 |  | 37.0 | 11.6 |
| Kal4.C22 | 15.0 | 32.4 |  | 14.1 |  | 28.3 | 13.4 |  |  | 47.5 | 11.6 |
| Kal4.C42 | 17.9 | 37.6 |  | 12.4 |  | 29.5 | 16.0 |  |  | 32.1 |  |
| Kal5.A4 | 15.0 | 35.3 |  | 14.1 |  | 28.3 | 13.4 |  |  | 47.5 | 11.6 |
| Kal5.B2 | 15.0 | 35.1 |  |  |  | 29.3 |  |  | 21.2 | 45.3 |  |
| Kal5.C3 | 15.2 | 37.0 | 13.4 | 14.8 | 18.2 | 33.0 |  |  |  | 45.1 |  |
| Kal5.C4 | 15.0 | 35.3 |  | 10.2 | 17.9 | 32.3 |  |  |  | 20.1 |  |
| Kal5.C5 | 12.5 | 37.1 |  | 15.3 |  | 28.4 |  |  | 11.5 | 43.1 |  |
| Kal5.C6 | 15.0 | 35.3 |  | 10.2 | 17.9 | 32.3 |  |  |  | 20.1 |  |
| Kal5.C7 | 15.0 | 35.3 |  | 10.2 | 17.9 | 26.3 |  |  |  | 20.1 |  |
