# Supplementary material for: Diversity of the var gene family of Indonesian Plasmodium falciparum isolates
Source: Malar J. 2013 Feb 27;12:80. doi: 10.1186/1475-2875-12-80 (PMC3614516; doi:10.1186/1475-2875-12-80)
Supplement: Additional file 7 — Distribution of homology blocks in cys2 sequences from DBL1a domain, classification by Rask et al. using VarDom server. Description: The table shows the distribution of homology blocks (HB) in DBL1a sequences containing two cysteine residues (cys2) using varDom server. Almost all cys2 sequences contained HB3 and HB5 as major homology blocks. HB60 was present in all cys2 sequences but no HB36 was found in any cys2 sequence. HB14 was present in both cys4 and cys2 sequences. Sequences from severe malaria cases, gDNA (white letters). Sequences from severe malaria cases, cDNA culture (orange letters). Sequences from severe malaria cases, cDNA filter paper (yellow letters on red). Sequence from an uncomplicated case, cDNA filter paper (yellow letters on black). Sequences from uncomplicated malaria cases, gDNA (black letters). [file 1475-2875-12-80-S7.doc]

**Additional Table 5. Distribution of homology blocks (HB) in** **cys2 sequences from DBLα domain (classification by Rask *et al*. using VarDom server)**

| Sequence | Score  HB3 HB5 HB2 | | | Score S2b | | | | | Score S2c | | | |
| --- | --- | --- | --- | --- | --- | --- | --- | --- | --- | --- | --- | --- |
|  |  |  |  | HB64 | | HB54 | HB14 | HB79 | HB131 | HB60 | HB36 | HB88 |
| Pap1.A3 | 15.2 | 29.9 |  |  | |  | 27.8 |  |  | 38.1 |  |  |
| Pap1.A4 | 15.2 | 36.7 | 11.8 | |  |  | 30.7 |  |  | 48.2 |  |  |
| Pap1.B3 | 15.0 | 33.0 |  |  | |  | 27.4 |  |  | 41.5 |  |  |
| Pap1.10 | 15.0 | 37.8 |  |  | |  | 13.0 |  |  | 35.3 |  |  |
| Pap1.11 | 15.2 | 35.9 |  |  | | 12.7 | 26.6 |  |  | 33.9 |  |  |
| Pap1.12 | 15.2 | 35.9 |  |  | | 12.7 | 11.5 |  |  | 33.9 |  |  |
| Pap1.14 | 15.2 | 35.9 |  |  | | 12.7 | 26.6 |  |  | 33.9 |  |  |
| Pap1.31 | 15.2 | 40.0 |  | 14.8 | | 20.5 | 37.5 |  |  | 33.6 |  |  |
| Pap1.32 | 15.2 | 36.3 | 11.8 | |  | 12.7 | 26.6 |  |  | 33.9 |  |  |
| Pap1.FP |  | 35.9 |  | |  | 11.6 | 26.6 |  |  | 33.9 |  |  |
| Pap2.C1 | 19.2 | 36.8 |  | | 17.5 | 17.6 | 29.6 |  |  | 21.6 |  |  |
| Pap2.C2 | 18.1 | 36.4 |  | |  | 11.2 | 26.1 |  | 18.2 | 47.7 |  |  |
| Pap2.C3 | 19.4 | 36.4 | 13.4 | |  | 11.2 | 26.1 |  | 18.2 | 47.7 |  |  |
| Pap2.C4 | 19.4 | 33.1 |  | |  | 11.1 |  |  |  | 35.4 |  |  |
| Pap2.FP | 15.0 | 36.3 | 13.4 | |  |  | 25.5 |  |  | 35.9 |  |  |
| Pap3.A1 | 15.0 | 38.2 |  | |  |  | 21.8 |  |  | 44.8 |  |  |
| Pap3.A2 | 15.0 | 40.3 |  | |  |  | 24.4 |  |  | 19.4 |  |  |
| Pap3.A8 | 15.0 | 29.5 |  | |  | 13.3 |  |  |  | 34.2 |  |  |
| Pap3.A9 | 15.0 | 29.5 | 13.4 | |  | 13.3 |  |  |  | 34.2 |  |  |
| Pap3.A10 | 15.0 | 29.5 |  | |  | 13.3 |  |  |  | 34.2 |  |  |
| Pap3.C8 | 15.0 | 40.1 |  | | 11.1 |  | 28.0 |  |  | 42.7 |  |  |
| Pap3.C9 | 15.0 | 40.1 |  | | 11.1 |  | 28.0 |  |  | 42.7 |  |  |
| Pap3.FP |  | 40.1 | 18.8 | | 11.1 |  | 28.0 |  |  | 42.7 |  |  |
| Kal1.A2 | 15.0 | 34.8 |  | |  | 11.5 |  |  |  | 39.2 |  |  |
| Kal1.B3 | 18.1 | 38.4 |  | | 15.4 | 19.5 | 30.2 |  |  | 37.1 |  |  |
| Kal1.FP |  | 37.4 |  | |  |  | 19.1 |  |  |  |  |  |
| Kal2.B2 | 15.0 | 35.1 | 13.4 | |  |  | 29.2 |  |  | 44.4 |  |  |
| Kal2.FP |  | 37.4 | 14.5 | |  |  | 27.3 |  |  | 43.5 |  |  |
| Kal3.F7 | 17.9 | 37.0 |  | |  |  | 22.7 |  |  | 39.1 |  |  |
| Kal3.F10 | 17.9 | 38.6 |  | |  |  | 24.6 |  |  | 40.8 |  |  |
| Kal4.C62 | 17.9 | 34.5 |  | |  |  | 15.5 |  |  | 35.2 |  |  |
| Kal5.C1 | 15.0 | 34.5 | 13.4 | |  |  | 15.5 |  |  | 43.7 |  |  |
| Kal5.C2 | 15.0 | 37.4 |  | |  |  | 27.3 |  |  | 43.7 |  |  |
| Kal5.C8 | 15.0 | 37.4 |  | |  |  | 27.3 |  |  | 43.7 |  |  |
| Kal5.FP | 15.0 | 36.3 | 13.4 | |  |  | 25.5 |  |  | 35.9 |  |  |
