# Supplementary material for: Diversity of the var gene family of Indonesian Plasmodium falciparum isolates
Source: Malar J. 2013 Feb 27;12:80. doi: 10.1186/1475-2875-12-80 (PMC3614516; doi:10.1186/1475-2875-12-80)
Supplement: Additional file 8 — Motif distribution of expressed var DBL1α Sequences. Description: The table shows the distribution of expressed var DBL1α sequence motifs (cysteine/PoLV sequence grouping classification by Bull and colleagues). All sequences extracted from blood from filter paper and therefore expressed in the patient during infection showed group 1-3. [file 1475-2875-12-80-S8.doc]

**Additional Table 6. Distribution motif of expressed *var* DBL1α sequences**

| **RNA Source** | **Cysteine/PoLV grouping (cys2)** | | | **Cysteine/PoLV grouping (cys4)** | | **Cysteine/**  **PoLV grouping (cys others)** |
| --- | --- | --- | --- | --- | --- | --- |
|  | **Group 1**  (sequence) | **Group 2**  (sequence) | **Group 3**  (sequence) | **Group 4**  (sequence) | **Group 5**  (sequence) | **Group 6**  (sequence) |
| **Filter paper** | **3** | **0** | **2** | **0** | **0** | **0** |
| **Culture** | **0** | 1 | **2** | **3** | **0** | **0** |
