# Supplementary material for: Diversity of the var gene family of Indonesian Plasmodium falciparum isolates
Source: Malar J. 2013 Feb 27;12:80. doi: 10.1186/1475-2875-12-80 (PMC3614516; doi:10.1186/1475-2875-12-80)
Supplement: Additional file 10 — Sequence family classification of DBLβ-C2 domain from field isolates using varDom server. Description: The table shows the score for sequence family classification of the DBLβ-C2 domain from field isolates using varDom server. All sequences were classified as Duffy-binding domain with a high score, more than 255.3 (threshold value 9.97), and all sequences except for DBLβ-C2 domain of Kal2 isolate were classified as DBLβ domain with a score more than 703.1, Kal2_ DBLβ-C2 domain is a non-typical Duffy binding domain. [file 1475-2875-12-80-S10.doc]

**Additional Table 7.** **Sequence family classification of DBLβ-C2 domain from field isolates using varDom server**

| **DBLβ-C2 sequence** | **Score for sequence family classification** | |
| --- | --- | --- |
|  | **Duffy-binding domain** | **DBL β domain** |
| Pap1 | 345.0 | 719.0 |
| Kal1 | 393.1 | 858.0 |
| Pap2.1 | 384.2 | 855.4 |
| Pap2.2 | 384.1 | 855.6 |
| Kal2 | 255.3 | - |
| Pap3.1 | 357.6 | 703.1 |
| Pap3.2 | 368.6 | 823.7 |
| Kal3 | 383.7 | 844.6 |
| Kal4 | 377.3 | 784.7 |
| Kal5 | 380.3 | 832.6 |
