# Supplementary material for: Diversity of the var gene family of Indonesian Plasmodium falciparum isolates
Source: Malar J. 2013 Feb 27;12:80. doi: 10.1186/1475-2875-12-80 (PMC3614516; doi:10.1186/1475-2875-12-80)
Supplement: Additional file 11 — Distribution of five major homology blocks in DBLβ domains of field isolates using VarDom server. Description: The table shows the distribution of five major homology blocks (HB1 – HB5) in DBLβ domains of field isolates using varDom server. All sequences contained five major homology blocks except for Kal2 where HB1 was absent. [file 1475-2875-12-80-S11.doc]

**Additional Table 8. Distribution of five major homology blocks (HB) in DBLβ domains of field isolates using VarDom server**

| **DBLβ Sequence** | **Score HB4** | **Score HB3** | **Score HB5** | **Score HB2** | **Score HB1** |
| --- | --- | --- | --- | --- | --- |
|
| Pap1 | 31.8 | 37.2 | 39.7 | 37.2 | 23.7 |
| Pap2.1 | 36.6 | 33.4 | 37.8 | 37.2 | 25.5 |
| Pap2.2 | 36.6 | 33.4 | 37.8 | 37.2 | 25.5 |
| Pap3.1 | 31.6 | 34.8 | 36.7 | 37.2 | 33.8 |
| Pap3.2 | 33.0 | 37.3 | 37.5 | 37.2 | 21.5 |
| Kal1 | 34.3 | 37.4 | 45.7 | 37.2 | 23.4 |
| Kal2 | 32.9 | 34.6 | 44.8 | 20.6 | - |
| Kal3 | 34.3 | 37.4 | 45.7 | 31.4 | 23.4 |
| Kal4 | 34.3 | 37.4 | 45.7 | 37.2 | 23.4 |
| Kal5 | 34.3 | 33.4 | 45.7 | 37.2 | 11.3 |
