# Supplementary material for: Diversity of the var gene family of Indonesian Plasmodium falciparum isolates
Source: Malar J. 2013 Feb 27;12:80. doi: 10.1186/1475-2875-12-80 (PMC3614516; doi:10.1186/1475-2875-12-80)
Supplement: Additional file 12 — Distribution of homology blocks among the proposed var D gene using VarDom server. Description: The table shows the distribution of homology blocks (HB) among the proposed var D gene using varDom server. The two motifs HB3 and HB5 seemed to be conserved features. Only one sequence shared HB86 with the previously described var D gene (AJ277137), where HB86 is mainly found in the DBLγ domain. Other sequences presented either HB82, HB98, HB27 or HB624. HB82 and HB98 are found in both DBLγ and DBLδ, while HB27 is mainly found in DBLδ. [file 1475-2875-12-80-S12.doc]

**Additional Table 9. Distribution of homology blocks (HBs) among the proposed *var* D gene using VarDom server**

| **Sequence** | **Score HB3** | **Score S2b subdomain** | | | | | **HB5** |
| --- | --- | --- | --- | --- | --- | --- | --- |
|  |  | **HB82** | **HB86** | **HB98** | **HB27** | **HB624** |  |
| Pap1.*var* D-like | - | 26.5 | - | - | - | - | 16.4 |
| Pap2.*var* D-like | 24.6 | - | - | - | - | - | - |
| Pap3.*var* D-like | 31.3 | - | - | 30.1 | 14.7 | - | 22.5 |
| Kal2.*var* D-like | 35.5 | - | 32.0 | - | - | 18.1 | - |
| AJ277137 (*var* D) | 37.4 | - | 32.6 | - | - | - | 32.2 |
